# Characterizing and Measuring Maliciousness for Cybersecurity Risk Assessment

**DOI:** 10.3389/fpsyg.2018.00039

**Published:** 2018-02-05

**Authors:** Zoe M. King, Diane S. Henshel, Liberty Flora, Mariana G. Cains, Blaine Hoffman, Char Sample

**Affiliations:** ^1^School of Public and Environmental Affairs, Indiana University Bloomington, Bloomington, IN, United States; ^2^Army Research Laboratory, Aberdeen Proving Ground, Aberdeen, MD, United States; ^3^Army Research Laboratory, Adelphi, MD, United States

**Keywords:** human risk factors, malicious intent, cyber security, cyber terrorism, rational behavior, metrics, motivation

## Abstract

Cyber attacks have been increasingly detrimental to networks, systems, and users, and are increasing in number and severity globally. To better predict system vulnerabilities, cybersecurity researchers are developing new and more holistic approaches to characterizing cybersecurity system risk. The process must include characterizing the human factors that contribute to cyber security vulnerabilities and risk. Rationality, expertise, and maliciousness are key human characteristics influencing cyber risk within this context, yet maliciousness is poorly characterized in the literature. There is a clear absence of literature pertaining to human factor maliciousness as it relates to cybersecurity and only limited literature relating to aspects of maliciousness in other disciplinary literatures, such as psychology, sociology, and law. In an attempt to characterize human factors as a contribution to cybersecurity risk, the Cybersecurity Collaborative Research Alliance (CSec-CRA) has developed a Human Factors risk framework. This framework identifies the characteristics of an attacker, user, or defender, all of whom may be adding to or mitigating against cyber risk. The maliciousness literature and the proposed maliciousness assessment metrics are discussed within the context of the Human Factors Framework and Ontology. Maliciousness is defined as the intent to harm. Most maliciousness cyber research to date has focused on detecting malicious software but fails to analyze an individual’s intent to do harm to others by deploying malware or performing malicious attacks. Recent efforts to identify malicious human behavior as it relates to cybersecurity, include analyzing motives driving insider threats as well as user profiling analyses. However, cyber-related maliciousness is neither well-studied nor is it well understood because individuals are not forced to expose their true selves to others while performing malicious attacks. Given the difficulty of interviewing malicious-behaving individuals and the potential untrustworthy nature of their responses, we aim to explore the maliciousness as a human factor through the observable behaviors and attributes of an individual from their actions and interactions with society and networks, but to do so we will need to develop a set of analyzable metrics. The purpose of this paper is twofold: (1) to review human maliciousness-related literature in diverse disciplines (sociology, economics, law, psychology, philosophy, informatics, terrorism, and cybersecurity); and (2) to identify an initial set of proposed assessment metrics and instruments that might be culled from in a future effort to characterize human maliciousness within the cyber realm. The future goal is to integrate these assessment metrics into holistic cybersecurity risk analyses to determine the risk an individual poses to themselves as well as other networks, systems, and/or users.

## Introduction

Cybersecurity is a critical and growing problem worldwide. During the timeframe in which this paper has been written, assets in at least 150 countries were hit with the WannaCry ransomware exploits released by the alleged “Shadow Brokers” with corrupted National Security Agency documents and files, and the Equifax data was breached, leaking personal and credit information for 143 million people (Bilefsky and Perlroth, 2017; Cherney, 2017; Thompson and Mullen, 2017). The current approach to cybersecurity risk assessment neither protects against unknown threats (e.g., WannaCry), nor does it consider the vulnerabilities introduced by humans using or interacting with the network, network defenders (IT professionals), or the attackers who intentionally introduce risk into the system. Historically, maliciousness as a cybersecurity phenomenon has been quantified in the technical expression of malware. To protect confidentiality of a network, IT professionals commonly use the National Institute of Standards and Technology (NIST) cybersecurity risk assessment model to recognize known system vulnerabilities and threats {listed in online databases such as NIST’s National Vulnerability Database [National Institute of Standards and Technology (NIST), 2017b]}, and, ideally, identify and install the best currently available protection software and hardware to address these threats [National Institute of Standards and Technology (NIST), 2017a]. The issue with this technique is that the NIST framework doesn’t consider human factors beyond using frequency of user data to help prioritize protections, meaning there is no realization that human behavior directly impacts cybersecurity risk. Our thesis is that maliciousness is a sociotechnical issue. The explicit integration of human factors into cybersecurity risk assessment is necessary to fully understand and characterize the impact of malicious behavior as it is reflected throughout all levels of society. The way people think and behave is just as important to study as the malicious code used to exploit vulnerabilities in technology.

**FIGURE 1:**
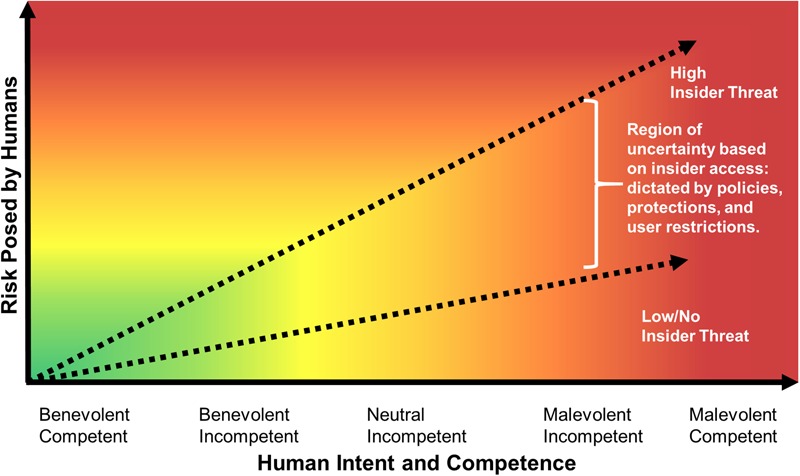
Visual representation of cyber security risk posed by humans given benign or malicious intent and level of computational competence.

**FIGURE 2:**
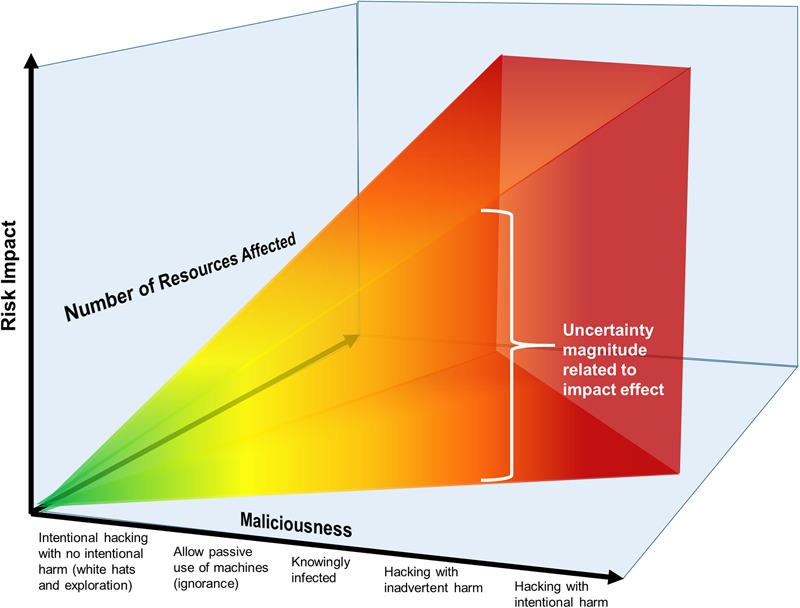
Visualization of risk posed by a human factor as a function of maliciousness-related cyber activity.

**FIGURE 3:**
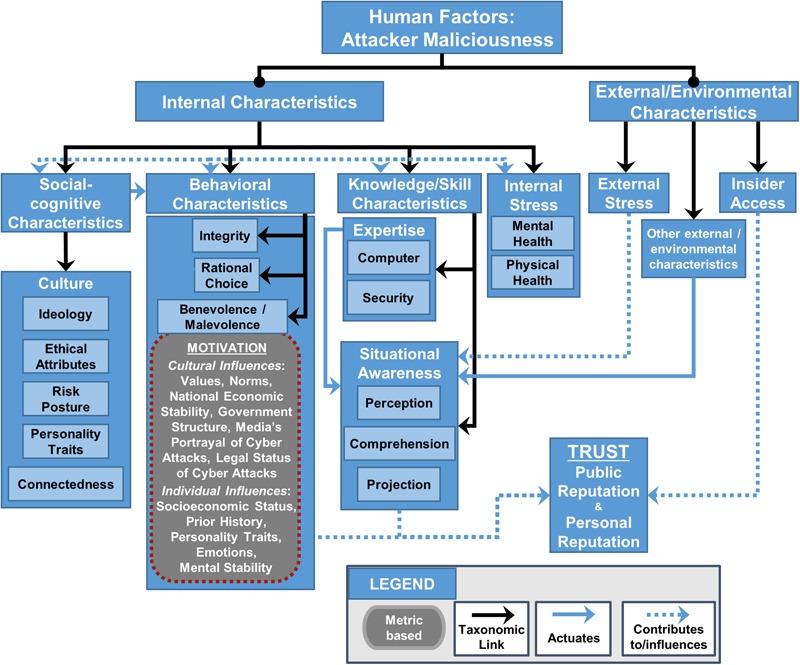
Human Factors Framework (modified from Henshel et al., 2015): Expansion of benevolence/malevolence assessment metrics to include malevolent behavior stemming from social cognitive characteristics, knowledge, and internal stress. This figure depicts how social-cognitive characteristics (i.e., those learned from culture) can influence behavioral characteristics such as malevolence.

Henshel et al. (2015, 2016) systematized human variables as risk factors within a holistic cybersecurity risk assessment framework (see **Figure 3** for a variant) for use in cybersecurity network risk modeling. Within the maturing Human Factors Framework and Ontology (and in practice), humans function as either risk inducers or risk mitigators (Oltramari et al., 2015; Henshel et al., 2016). As a part of developing this holistic risk model, we propose a new, focused characterization of humans (users, defenders, and attackers) from a cybersecurity perspective using a set of four factors that are scalable: rationality, expertise, malevolence or maliciousness, and insider access (**Figures 1, 2**). These four factors are included as risk variables attributed to humans (such as the attackers) in a fully parameterized risk assessment. This effort aligns with a highlighted essential improvement for cyber risk modeling in a recently released World Economic Forum report (World Economic Forum [WEF], 2015).

**Figure 1** depicts the relationship between the risk posed by humans and the human’s intent and competence within the cyber realm. This graph demonstrates that cybersecurity risk increases with attacker or insider threat maliciousness and insider access. Competence or skill influence on risk varies with the intent of the human. Competence reduces risk if the user or defender intends no harm (Benevolent), and introduces more risk than an unskilled or incompetent person if the human has neutral intent or is intentionally acting maliciously.

**Figure 2** depicts the relationship between types of hacking or cybersecurity risk-related activity, the relative number of resources affected, and the relative potential for risk to the network or network-accessed data. Hackers can have malicious intent, i.e., purposefully harm people or systems, or may have benevolent intent, i.e., to identify vulnerabilities in systems for purposes of ultimately reducing or correcting those vulnerabilities. The x-axis shows the range of different kinds of hacking-related activities, from the most benevolent toward the origin, to the most malicious away from the origin. Clearly, as malicious intent of hacking or invasive activity increases and more resources are vulnerable to the attack, the total risk of adverse impact increases too. Thus, the benevolence/malevolence of the intent behind any hacking activity is directly relevant to cybersecurity risk and it is important for a measure of maliciousness to be incorporated into cybersecurity risk models.

Maliciousness is defined as the intent to harm (Maliciousness, n.d.). Although cybersecurity expertise and rational behavior are well researched, malicious intent is currently understudied and therefore difficult to quantify and characterize from a cybersecurity or even terrorism perspective. (Terrorism, a related behavior, can potentially be addressed by the same methods of analysis.) Maliciousness is a characteristic of risk inducers (including overt attackers, hostile defenders and users intending harm to the [cyber] system with which they interact). It is when people are at their most malicious that they are most likely to harm another, act as a terrorist, or act as a cyber attacker, making maliciousness a key human parameter needed to characterize and quantify human risk factors in a cybersecurity risk assessment model. Maliciousness is both stochastic and dynamic; many people who act maliciously are not inherently malicious, and many people who are not generally malicious act maliciously toward others at some time in their lives. Further, maliciousness triggers are identifiable. For example, road rage is malicious intent evoked by some purposeful or inadvertent action by another driver (such as cutting someone else off). People may also carry out malicious intentions without ostensibly malicious motives, such as vigilantes seeking to right a (perceived) wrong. We consider these triggers to be a result of being stimulated and cognizant of that action. The initial impulse felt when triggered is an emotion, described later. The results of the literature review and proposed maliciousness assessment metrics that may be useful in developing a cyber maliciousness assessment instrument are addressed at multiple levels. We first assess the individual in terms of how they behave when not influenced by interpersonal, intergroup, or societal influence, then we consider individuals within the context of interpersonal, intergroup and societal influence. Thus, we address four levels of human factors related to maliciousness: individual, micro-, meso-, and macro-cultural factors.

In this paper, we review and constructively critique the literature pertaining to vectors and expressions of maliciousness in human behaviors from the perspective of how such behaviors might link to actions increasing cyber risk. We suggest that identification and classification of malicious human behavior is crucial to developing assessment and measurement metrics to classify defenders, users, and attackers according to their potential risk to cyber networks. Assessment metrics identify what needs to be assessed (Suter, 2006). Measurement metrics are the specific, measureable means by which assessment metrics are quantified. The best measurement metrics for quantifying cyber risk will be specific, measurable, accurate and achievable, relevant and reproducible, and quantifiable within the timeframe needed for the analysis (S.M.A.R.T.; Doran, 1981). While measurement metrics are explicit means to quantify the assessment metrics, assessment metrics may be quantified by one or a suite of measurement metrics. While the present paper does not develop a means of quantifying each assessment metric, it does offer several metrics with the intention of developing future methods of quantification using these metrics.

## Materials and Methods

The search for characteristics contributing to cyber-related human maliciousness encompassed the fields of sociology, economics, law, psychology, philosophy, informatics, cyber terrorism, and cybersecurity. Each discipline offered unique perspectives on maliciousness. Using Google Scholar (Google, n.d.), we separated literature on malware from literature on human maliciousness by searching terms such as: “malicious-acting software,” “malware,” and “malicious cyber attacks.” We then incorporated individual metrics for human maliciousness by searching such terms as: “malicious intent,” “psychopathy,” “human maliciousness,” “malice,” “intergroup aggression,” “interpersonal aggression,” “schadenfreude,” and “intent to harm.” Since cultural values and biases influence the way people act in groups and in response to interpersonal interactions, we searched for cultural metrics, including: “cultural motivation,” “history of cyber attacks,” and “hacking culture.” Macro-cultural metrics were determined through an extensive literature review of evaluations of national cultures and subcultures over time and a tree-search from the website for Hofstede’s national culture metrics (Hofstede and McCrae, 2004; Hofstede, n.d.).

## Results

In order to develop both assessment and measurement metrics for cybersecurity-related maliciousness, the term “maliciousness” must first be described as a human characteristic that affects event outcomes. This paper proposes that maliciousness for attackers is a function of personality traits, mental instability, emotions, self-perception, attitudes, biases, interpersonal behavior, marginality, values (individual and subcultural), norms, national economic stability, government structure, media portrayal of cyber attacks, legal status of cyber attacks, and intergroup behavior. These maliciousness assessment metrics have been incorporated into to the Human Factors Framework and Ontology for cybersecurity risk assessment (**Figure 3**; Henshel et al., 2015, 2016). We examine these factors through the level of social organization in which they occur: the individual, which focuses on personality and mental processes, the micro-level which covers interpersonal interactions, the meso-level which encompasses group membership and subcultures, and the macro-level in which we analyze the impact of belonging to large or national cultures.

**Figure 3** is an extraction of the Human Factors Framework (Henshel et al., 2015, 2016) that focuses on the assessment metrics, and potential measurement metrics that can be used to quantify the assessment metrics, linked to malicious intent for attackers. The Human Factors Framework and Ontology (Oltramari et al., 2015) organizes the hierarchical connections between the assessment and measurement metrics by which these factors contribute to public and personal trust in an individual – in this case, of an attacker. Internal characteristics are characteristics inherent to the individual, while external/environmental characteristics are sourced outside the individual. Not all assessment and measurement metrics referred to in **Figure 3** will be discussed in this paper, but all have potential relevance to quantifying malicious intent and malicious activity.

### Metrics Associated with Malicious Behavior

All metrics, assessment level (like Social-Cognitive Characteristics) or measurement level (like personality traits such as the Dark Triad traits), exist at one of four levels of social organization, as discussed above. Thus the following discussion organizes the literature by individual, micro-, meso- and macro-metrics.

#### Individual Metrics Associated with Malicious Behavior

Metrics driving the intent to harm on an individual level include those influenced by personal characteristics as well as those influenced by culturally defined values, norms, and biases. Rational choice is how an individual ranks potential outcomes of events in terms of benefit versus cost. Socioeconomic status is relevant because motivations change depending on the amount of money a person has. Within a particular socioeconomic group, power dynamics and perceived isolation may also influence motivation to act maliciously. Individual metrics associated with malicious behavior were derived with the assumption that individuals have characteristics inherent within them. The individual metrics do not take into account social interactions as a basis for behavior. Certain personality traits can increase the risk of a particular malicious act, because personality traits are tendencies to behave a certain way in a given environment (Lawrence et al., 1995/1999). Mental instability is anything that disrupts rational thought. An example of mental instability is Psychopathy, and we discuss this (below) in terms of how particular personality traits related to Psychopathy put someone at a higher risk of acting maliciously. Finally, we propose prior history correlates to behavioral disposition.

#### Micro-Level Metrics Associated with Malicious Behavior

Metrics associated with the intent to harm on the micro level include those influenced by both personal characteristics as well as culturally biased values, norms, and biases. This level of interaction involves interpersonal relationships through which an individual is taught to think and act a certain way by means of learning from experience (LoBue et al., 2010). Emotions sparked by interpersonal interactions may motivate an individual to behave maliciously. An individual’s prior history, such as a traumatic experience, may be associated with maliciousness because a person may alter their self-perception or their social environments differently than before the experience. The perception of social environments is referred to as schema, which is impacted by attitudes and biases. Both attitudes and biases are determined as a result of past experiences. We discuss self-perception as a result of marginality, which occurs as a result of both interpersonal and intergroup relationships.

#### Meso-Level Metrics Associated with Malicious Behavior

A large number of meso-level factors are associated with human maliciousness as well as the deployment of malicious software by humans. Subcultural values, or the moral principles of a subculture, affect how an individual will perceive the world and influence individual behaviors. Once a part of a peer group, individuals may act maliciously based on their group’s relationship with others. Therefore, intergroup behavior may be an important factor associated with meso-level maliciousness. Individuals may be inclined to join malicious-acting peer groups when identifying with any of the following factors: low socioeconomic status, socialization toward rule-breaking behavior, hyperactivity, family adversity, being of the male gender, and limiting one’s social behavior to the peer group (Lacourse et al., 2006). Cyber attackers have also identified factors that led them to partake in groups or subcultures that performed malicious cyber attacks. These factors include: smart children’s boredom with inadequate schools, schools’ inability to notice the children’s differences, and the desire to act out against corporations that profit from overpriced web services (Blankenship, 1986; Yar, 2005). Further, social scientists believe men may join deviant groups of hackers when they are frustrated with women or want to assert their masculinity (Yar, 2005).

#### Macro-Level Metrics Associated with Malicious Behavior

The following relevant cultural assessment metrics influence human behavior: values, norms, national economic stability, government structure, media’s portrayal of cyber attacks, and the legal status of cyber attacks. *Values*, as moral judgments, play a large role in framing specific attacks as good or bad, which may motivate individuals to perform, support, or condemn an attack (Markus and Kitayama, 1991). *Norms*, or the customary behaviors of a group, contribute to the separation of culturally sanctioned and deviant motives and also pressure the individual to behave in a particular manner (Morgan et al., 2015). *Intergroup behavior* contributes to collective perceptions of outgroups (Smith et al., 2015). Negative perceptions of outgroups coupled with strong in-group affiliation can influence individuals to act maliciously toward outgroup members. *National economic stability* generally affects public perception of the government and can affect the general public’s support or criticism of cyber attacks against the government (Burke et al., 2015). *Forms of government* influence public and personal approval and may contribute to an individual’s motivation to act for or against their government (Burke et al., 2015). *Political stability*, in terms of government reliability and average satisfaction with the government, is also likely important (Burke et al., 2015). The *media’s portrayal of cyber attacks* as either malicious or benevolent influences individuals to act according to perceptions depicted by newscasters (Knapp, 2014). The *legal status of cyber attacks* also plays a large role in cyber attacks, allowing individuals to weigh the legal costs of performing an attack and influencing public perception by assigning penalties that suggest the severity of attacks (Wilmes, 2006).

### Different Schools of Thought and Controversies

Cybersecurity research has historically focused on malicious-acting software to represent attackers. In modeling cyber threat risk, human factors are often overlooked due to their difficulty of analysis and lack of accessible data. For accuracy in determining when an individual is likely to behave maliciously, it would be ideal to characterize each and every individual and make note of patterns in individual attributes increase their risk of behaving maliciously. However, this approach is simply not feasible. It is also not right to assume that individual (inherent) characteristics or (on the other extreme) that culture is the sole driver of maliciousness. Rather, we are proposing an individual will learn from their interactions with others such that their future behavior will be influenced. The goal is to be able to identify patterns in the intensity of influence of personal attributes coupled with multiple levels of interaction.

In the following section, we explain the current state of cybersecurity research in order to emphasize its overwhelming focus on malware as well as demonstrate the limited approaches to incorporating human factors. We then address how research that focuses solely on software falls short in its analysis of cyber threat.

#### Malware to Represent Human Maliciousness

In the cybersecurity literature, the notion of maliciousness is most frequently associated with malware, a portmanteau of malicious software. Cybersecurity articles, blogs, and malware reports emphasize the concept of malware as “malicious code.” Malware is not commonly thought of in terms of the human actors involved. Rather, maliciousness is characterized by and attributed to the behavior of the malware, the types and orders of system calls, and the system permissions they require (Siddiqui et al., 2008; Sami et al., 2010; Canfora et al., 2013; Chia-mei and Gu-hsin, 2014). Cyber attackers are not in the habit of announcing themselves ahead of time, and network defenders rely on suites of tools, including intrusion detection systems (IDS), anomaly behavior detection tools, firewalls, and signature-based or heuristics-based detection techniques to try to find and characterize malware before the malware inflicts harm upon a system, network, or user. Attackers may spoof and hop through pivot points to obfuscate the origin of attacks and make it difficult for accurate attribution. However, IP addresses and similar information gathered via packet inspection affords a starting point to reason about possible characteristics of the source of an attack, and forensic analysis of malware affords a chance to explore how it works and its goal(s). For example, certain coding styles or features could provide clues to the mindset or characteristics of the individual(s) who crafted it.

Despite the prevalence of botnets and automated attacks, research has shown that there are bursty patterns and non-random exploitations of vulnerabilities, indicators of the human actors who, through expertise, demonstrate capability and intent to employ cyber attacks (Barabasi, 2005; Gil et al., 2014; Liu et al., 2015). Cyber attacks are being crafted and deployed in systematic, coordinated efforts that follow the objectives of malicious actors; effective deterrence hinges on understanding these actors (Jasper, 2015). On some occasions cybersecurity research has focused on human actors. Often, this research has sought to identify a taxonomy of different types of attackers. For example, research on maliciousness in crowdsourcing platforms has identified four types of workers through a survey: fast deceivers, rule breakers, smart deceivers, and gold standard preys (Gadiraju et al., 2015). Other research has identified two types of insider attackers: masqueraders, who steal another user’s identity for malicious purposes, and traitors, who are legitimate users with access to certain networks but who act maliciously (Salem et al., 2008). Furthermore, research on end-user behaviors has identified an important taxonomy of the interplay between intent and expertise. They classify the behaviors of end-users with high expertise and malicious intentions as intentional destruction while end-users with high expertise and benevolent intentions are said to perform acts of aware assurance. In addition, end-users with low expertise can either act maliciously, resulting in intentional misuse, or beneficially, resulting in basic hygiene (Stanton et al., 2005). Therefore, it is important to consider intent, behavior, and skill set when profiling attackers.

#### Issues with Malware Research

While some studies have focused on human factors associated with malicious-acting software, many have chosen to forego the human factor method of analysis and focus mainly on malware. We argue that understanding and characterizing human factors driving the deployment of malicious software are extremely important in quantifying cyber threat. The incorporation of human factors has been a source of debate in cybersecurity (Henshel et al., 2016). Some researchers believe it is difficult to quantify due to its seemingly limitless myriad of potential factors; others may protest its use in cybersecurity as we have little knowledge about the humans who perform cyber attacks since they often remain unidentifiable. We argue here for the school of thought in which human factors must be explicitly integrated into cyber threat risk assessment.

### Fundamental Concepts, Issues, and Problems: Incorporating Human Factors

Multiple human factors associated with maliciousness may lead someone to act maliciously. Numerous psychological and sociological studies have associated individual and cultural traits with malicious intent, motivation, and/or behavior at the individual, micro, meso, and macro levels of analysis. We draw from studies in each of the aforementioned sections to create a series of metrics that we believe will help characterize human maliciousness related to cyber threat.

#### Malicious Intent

Malicious intent, as defined by The American Law Institute, is when an actor desires to inflict harm or believes negative or adverse consequences will result from his actions (Jung and Levine, 1986). Maliciousness is simply defined as the intent to harm. Intent includes desire to act as well as the decision of whether or not the act will occur. Malicious intent is related to criminal intent, as defined in American culture, by means of a guilty mind (mens rea; Maasberg et al., 2015). Under this assumption, malware intentionally employed to damage or destroy a computer system is inherently malicious, regardless of the motive(s) driving the attack. Therefore, the intentional deployment of malicious software by human actors is linked to human maliciousness. There is ambiguity in determining what behavior is malicious and what is benign because the intent may be perceived as both malevolent and benevolent depending on the cultural, social, and individual acceptance of such behavior. The ultimate goal of an individual determines benign versus benevolent behavior. Benevolence is associated with compassion (Benevolence, n.d.), whereas benign behavior, in terms of cyber hacking-related activities, occurs when an individual intends to gain benefits but not at the cost of others (Verizon Risk Team, 2011). Malicious intent, while subjective in terms of identifying whether or not someone truly intends to do harm, could be identified if the motivation driving the attack is known. We will discuss motivation and its relationship to intent, but we also emphasize that analyzing motivations is not the most effective method of determining the intent to harm.

#### Motivation for Malicious Behavior

Intent focuses on the determination and state of mind to carry out an act, while the term “motive” describes the reasons an individual may act (Maasberg et al., 2015). Malicious motives can include financial gain, political gain, personal gain, competitive advantage, and revenge as well as result from sacred beliefs (Frijda, 1996; D’Errico et al., 2015). There is no ubiquitous motivational factor driving malicious intent; rather, we believe there is a wide array of motivations driving an individual’s intent. While motives play a large role in human behavior, in the context of maliciousness and cyber security they are even more unidentifiable than the human behind the computer screen. Therefore, motivations driving the intent to harm are nearly impossible to quantify (Maasberg et al., 2015). Therefore, we focus on factors that have historically correlated with malicious behavior. However, we make no attempt at establishing a causal relationship between malicious behavior and the factors described.

### Individual, Micro, Meso, and Macro Humans Factors Associated with Maliciousness

The human factors associated with maliciousness and cyber attacks vary greatly; for organizational purposes, as above, we have divided the factors by level of analysis starting at the individual and micro (interpersonal) scales, and then continuing to the meso and macro levels to delineate the cultural traits associated with maliciousness and cyber attacks.

#### Individual Factors

As individuals, we believe what is right and what is fair, and commonly make choices to coincide with those beliefs (Power and Dalgleish, 2015). How individuals decide to act on these beliefs determines whether or not they are acting maliciously toward others. At the individual level, personality traits are the main factors that contribute to malicious behavior.

In order to classify common personality traits and predict online behaviors, many researchers turn to taxonomical classification. Personality is recognized by prototypical traits, which are long-lasting, intrinsic attributes of an individual and are not a result of interactions with others. The “Big Five” personality traits (openness to new experiences, conscientiousness, extraversion, agreeableness, and emotional stability) are recognizable personality traits in both the cyber and physical realms (Lawrence et al., 1995/1999). There is some concern that the Five Factor Model, in which the Big Five personality traits are incorporated, does not account for individual differences in personality traits, such as antisocial behavior. The Dark Triad traits are believed to be a better representation of personality characteristics amongst malicious individuals than the Big Five personality traits. The Dark Triad Traits (psychopathy, narcissism, and Machiavellianism) are commonly examined to identify characteristics of employees who are known to have employed malicious attacks or contributed to a malicious act (e.g., insider threats; Maasberg et al., 2015). Being able to characterize personality traits on a large scale is useful and less time consuming than examining the motives driving a single individual’s intent to harm. However, social experiences and interactions in which a person exemplifies their personality traits toward others are necessary to understand why a person is inclined to behave a particular way.

#### Relating the Individual to Higher Levels of Interaction

Maliciousness, as defined by the intent to harm, is inherent in any intentional deployment of malicious-acting software, since this software is used to cause harm to a system. However, humans may be motivated to perform cyber attacks for a number of reasons; despite harming a computer system, humans may perceive malicious cyber attacks as beneficial to the group with which they associate themselves. As perceptions of malicious cyber attacks may vary depending on the group, culture plays a large role in human maliciousness. Culture helps influence human behavior as it constitutes a specific learning environment with particular norms, values, and beliefs that individuals may internalize to help guide their actions (Akers and Jensen, 2006). According to social learning theory, humans are anticipatory creatures whose motivations to perform any given behavior are contingent on the perceived, expected consequences of the behavior (Bandura and Walters, 1977). Because consequences are socially determined, positive and negative reinforcements for behavior vary with the culture (Akers and Jensen, 2006). Further, when determining how to behave, humans respond to decision tasks based on the way the task and its consequences are presented to an audience (Selten, 1998). The cultural framing of a task refers to the way specific cultural factors determine how the task will be presented to subjects. Cultural frames define problems in terms of cultural norms and values, guide moral evaluations, and provide solutions (Entman, 1993); they are established through laws, values, norms, beliefs, consequences, and other aspects of the group (Benford and Snow, 2000). Social learning environments, or cultures, provide unique frames that influence how someone will view a task and the positive or negative sanctions they will receive for performing the task. In the case of cyber attacks, any malicious attack or piece of malware may be framed as good or bad depending upon cultural factors (Kahneman, 2003). Individuals may internalize this framing and behave accordingly. Therefore, it is important to look at how cultural traits and group membership have influenced human maliciousness as well as cyber attacks.

#### Micro-Level of Interaction

Behavioral variation exists both between and within social groups (Muftić, 2006). The micro level of analysis focuses on individuals and their interactions with other individuals. Individuals may deviate from the norms and values of their larger culture and act maliciously despite the potential of receiving negative sanctions. Malicious motives form when individuals’ interactions with their surroundings create conflict (Verwimp et al., 2009). In these situations, socially acceptable behaviors may be compromised due to the perception that an interpersonal interaction was unfair or aggressive (Hewig et al., 2011). In the cyber realm, micro-level interaction may result in relatively benign behaviors, such as sharing mp3 files, or relatively deviant crimes like cyberbullying (Denegri-Knott and Taylor, 2005). The micro-level plays a role in determining individual behavior by incorporating cultural influence as well as person-to-person interactions, which are influenced by emotions. How a person perceives themselves and others, and how perceptions allow someone to develop biases and attitudes also ultimately influences behavior.

##### Emotions

Emotions offer cues into how a person feels before making the choice to act. A person is stimulated by and is cognizant of others’ behaviors, gestures, beliefs, desires and facial expressions (Power and Dalgleish, 2007). When humans observe others they often have an impulse to behave. Power and Dalgleish (2007) suggest the initial impulse is a “primary emotion,” which is an “approach to or a recoil from good or bad.” It is at this point in which a person undergoes a secondary emotion, and they have the choice of whether or not to act (Frijda, 1996). A series of secondary emotions occurs when an individual is inclined to behave a particular way toward others (Power and Dalgleish, 2007).

Emotions analysis may help predict whether or not an individual will act maliciously while online. The frustration-aggression theory states that although some aggression is impulsive, other forms of aggression consist of thinking and planning, meaning impulses and inclinations do not drive all behaviors (Smith et al., 2015). Emotions are also characterized by intensity (Frijda, 1996). Just because a person feels a particular way doesn’t mean they feel strongly enough to act on it. We consider emotions to be correlated with an individual’s behavior, but do not believe all people will feel or act the same way when in the same situation. Rather, we consider why a person is influenced strongly enough to behave maliciously.

##### Individual perspective of self

An individual creates an image of “self” based on how he or she thinks of him/herself, but also on how others perceive him or her (Turner, 1982). In creating this intrinsic image, the individual may evaluate how he or she is perceived to “fit” in terms of group-belongingness and social identification in order to uncover the means by which a goal can be obtained (e.g., moving with the help of friends versus doing it all alone). This may begin with a simple questions, such as, “Who am I?” From here, a person may ask questions such as: Who do I want to be? Who do I want to surround myself with? How do I attain my goals? These questions influence the means by which someone attains their goals, ultimately affecting behavior. Lawrence et al. (1995/1999) suggest individuals can be characterized by the traits they display over time, their typical internal state of well-being, physical states, the activities they are involved in, the influence they have on other individuals, social evaluation, and also by the roles they play.

##### Schema and its relationship with attitudes and bias

A schema is an individual’s internal representation of the social environment based on knowledge and understanding of experiences and events taking place in the past. It is based on an organization of perceptions rather than the objective features of the social environment. Schemas influence behavior by establishing the way future social events are perceived by an individual. The understanding of schemas is consistent with the concept of interpersonal behavior (Lease and Axelrod, 2001). Biases result from a stimulus and learning events that cause an individual to be cognizant of his or her negative emotions, such as fear (LoBue et al., 2010). A schema as a representation of the environment, when combined with biases, influences the way future social interactions are perceived. This behavioral influence, a result of previous experience, is correlated with social psychological theories of interpersonal behavior (Lease and Axelrod, 2001). Attitudes, on the other hand, are viewpoints regarding an event or object. Attitudes generally become more strongly felt with age and are relatively stable with one’s core beliefs (Ajzen, 2001). The question, however, is how to properly identify a malicious person who is very good at hiding emotions and beliefs (i.e., values). For example, all of the Dark Triad traits describe a person who has “emotional coldness,” meaning malicious intent may be well-hidden by an individual through manipulation of how others perceive him or her. Hiding true feelings is another example of why it is not feasible to only analyze the individual. We must consider the individual’s context, the bigger picture, i.e., cultural influence, when assessing whether or not an individual is likely to behave maliciously.

##### Interpersonal aggression

It is common to see the term “aggression” in literature examining malicious behavior. Aggression can take on multiple forms, such as hostile or instrumental aggression. Both hostile and instrumental aggression involve a single act which can be categorized as either interpersonal or intergroup aggression, depending on who is the out-group (Smith et al., 2015). Aggression can either be direct or indirect in all cases of physical, cyber, and/or psychological attacks (Fluck, 2014).

Interpersonal aggression is a general term to describe one’s desire to hurt another due to a threat to self-esteem or a feeling of disrespect, both of which are stimuli leading to emotion, desire, and occasionally behavior (Smith et al., 2015). Interpersonal aggression is the person-to-person altercation in which many types of malicious attacks are rooted; it forms on the basis of social identification, which is rooted in self-perception, how others perceive you, and how you think others perceive you (Turner, 1982). Cases of interpersonal aggression often involve people of marginal sociometric status, thus setting these individuals up for rejection across social groups (Wyatt and Haskett, 2001).

*Cyberbullying.* Interpersonal aggression can be extended to include cyber abuse like cyberbullying, which is a common form of relational or indirect aggression (Bentley et al., 2014). According to Mishna et al. (2009), cyberbullying occurs when an individual uses some form of technology (Internet sites, text messages, email, social media) to threaten, harass, embarrass, or destroy the social standing of another individual in an act of fear or vengeance. Unlike traditional bullying, the actor does not need to have physical power over his or her victim because it is an indirect interaction. In many cases of cyberbullying, the actor struggles with psychosocial issues such as complexes with parents, substance abuse, and delinquency (Mishna et al., 2009).

Fluck (2014) used a Confirmatory Factor Analysis (CFA) to quantify and compare results of a survey given to 578 middle-school children in Germany in order to develop theoretical assumptions about the motivations driving the intent to cyberbully. Fluck uses a five-point Likert scale to compare student survey answers with a Taxonomy of Reason (TOR) model. The TOR model categorizes aggressive acts based on five dimensions of motivators: instrumental, power, sadism, ideology and revenge; however, TOR lacks motivators such as enjoyment or curiosity. Fluck concluded that offenders are likely to justify their actions as some kind of necessary response to being hurt by someone else.

A subset of cyberbullying is rumor-spreading, which is indirect rather than direct aggression. Rumor spreading is common when an individual desires to destroy the social status of another individual (Lansford et al., 2012). Mediators, such as school programs to strengthen relationships between individuals (in order to reinforce prosocial behavior) and efforts to encourage parental support of children, are useful in combatting cyber hostile situations (Wyatt and Haskett, 2001).

*Sexual solicitation.* In the 1957 court case, The Queen v. Neil, the Supreme Court of Canada defined a criminal sexual psychopath as an individual who lacks the power to control sexual impulses to a point in which they are likely to attack or inflict harm upon another person (Supreme Court of Canada, 1957). A criminal sexual psychopath is analogous to an online predator. Online sexual solicitation is a similar type of interpersonal aggression. Online sexual solicitation occurs when individuals coerce others into sexual acts through the use of online media. Online predators establish trust and confidence with their victims before initiating sexual activities. Children who lack social skills or experience are more likely to enter chat rooms and be involved in risky online behavior, such as sending personal information to unknown people (Wolak et al., 2013). The offenders are likely to target victims in a form of instrumental aggression which encompasses all forms of predation and is based on mastery needs, such as the desire to have nice things. Instrumental aggression is a result of evaluating costs versus benefits (i.e., rational choice; Smith et al., 2015). In this case, the predators are normally angry, impulsive, curious, or desire power (Wolak et al., 2013). For this reason, the risk of an individual acting maliciously will be, in part, a combination of an individual’s mental stability, emotions, rational choice, and personality traits.

*Marginality.* The degree of marginality relates to how a person is perceived within the social system. Sociometric status places individuals in one of three categories: central (typical), moderate, or marginal (atypical). Rejected (marginal) individuals are likely to be “psychologically distanced,” “shy,” or have a “unique set of hobbies” (Wyatt and Haskett, 2001). These types of individuals commonly display aggressive characteristics toward others, a typical form of intergroup aggression (Smith et al., 2015). Socioeconomic status (i.e., social class) is determined by wealth, education, and income, and also affects one’s perceived hierarchical standing within a society (Greitemeyer and Sagioglou, 2016). Both personal identity and social identity are considered when evaluating an individual’s position within society (Turner, 1982).

#### Meso-Level of Interaction

A group is any collection of people who share place, similar identity, culture, and social relations; thus, groups exist on many different levels (Fine, 2012). The meso-level of analysis seeks to examine group affiliations to understand how socialization may differ from interpersonal and country level influences. Although not necessarily distinct from the national or macro-culture, meso-cultures can provide nuanced socialization in the form of discrete norms and values which may manifest as differences in the human factors associated with malicious behavior. In the context of cyber attacks, the nuanced socialization that may occur on the meso-level can often be deemed deviant when compared to the larger culture, yet is accepted on the meso-level by group peers (Akers and Jensen, 2006).

##### Geolocational cultural differences

Within a nation, variations in regional cultures are evident. Regions vary in terms of values, norms, and politics (Baer et al., 1993). Individuals may internalize both the national and regional norms and values; thus, both macro and meso levels of analyses are crucial in understanding individual perceptions of cyber attacks and the human factors associated with them (Hayakawa, 2000). The social environment in which one lives has a significant effect on one’s resources and attitudes. Social environments may provide resources such as access to social, economic, and cultural capital, dictate social and personal preferences, and also help group members define and gauge success (Fine, 2012). Therefore, geographic location and regional culture may play a role in an individual’s perception of cyber attacks and the factors that may lead one to perform them.

##### Subcultures

At the meso-level, social structural variables (race, gender, socioeconomic status) interact with learning environments (family socialization, peer association) and create deviant subcultures. Subcultures develop through people with shared disadvantages; these groups have values, norms, and beliefs that are at odds with the macro-culture and therefore labeled as deviant (Akers and Jensen, 2006). Because these groups have their own learning environments in which nuanced norms and values create subcultural frames, acts of macro-cultural deviance may elicit positive consequences in the subculture. These consequences then reinforce macro-cultural deviance by rewarding it within the subculture and, in turn, motivate individuals to act maliciously. Subcultures are especially important in the case of cyber attacks, because in many countries hacking is considered to be part of a deviant subculture. Because solidarity increases at meso- and micro-cultural levels and strong group affiliation often motivates a high risk of activism, subcultures of hackers can produce powerful, collective incentives to perform cybercrimes (Fine, 2012).

*Risk factors for joining a deviant subculture.* Factors leading to the formation of and participation in deviant subcultures are meso-level risk factors when evaluating cyber attackers. Typical risk factors for participation in deviant peer groups include: low socioeconomic status, hyperactivity, limiting one’s social behavior to the peer group, family adversity, and being of the male gender (deviant peer groups are more typically comprised of men) (Lacourse et al., 2006). According to ethnographic studies of cyber attackers, these meso-level risk factors are distinct for cyber attackers. In the 1986 document *The Conscience of a Hacker*, the famous hacker, “The Mentor,” outlines several push factors for individuals to join subcultures of cyber attackers: smart children’s boredom with inadequate schools; schools’ inability to notice the children’s differences; and the desire to act out against corporations that profit from overpriced web services (Blankenship, 1986; Yar, 2005). Even today, Anonymous and other socially conscious groups have taken on similar ideologies (Vance and Helft, 2010). Yar (2005) notes that because hacking culture is often male-dominated and characterized by misogyny, a common push factor may be disdain or frustration with women. Additionally, male hackers may partake in such deviance as a way to assert their masculinity when the larger culture associates masculinity with mathematical and logical problem solving. However, less individualized approaches toward deviant hacking groups typically describe socialization toward rule-breaking behavior through peer association, both on and offline (Yar, 2005). Any of these factors coupled with computer expertise may motivate an individual to join a deviant hacking group (Yar, 2005).

##### We-ness and we-thinking

After accepting that culture defines the group, individuals will seek to understand their role in the group (Matusitz, 2014). How social identification is recognized arises from how we perceive and define ourselves (Nisbett, 2010) as well as how we are viewed as by others. Social identification provides an explanation for intergroup and intragroup behavior in terms of how social groups are categorized (Turner, 1982). Social identification is based on affiliation with informal social groups (e.g., nationality, sex, gender, political affiliation, religion), specific personal attributes (e.g., competence, personal tastes, personality traits), and how a person feels they relate to others. The importance of shared values in defining culture can be seen when social groups are connected via sacred values in which the term “we-ness” is incorporated into a relationship.

We-ness describes the circles from which people exclude others; this act, in turn, builds up the confidence of those in the circle and demoralizes outgroup members (Dimitrova et al., 2010). In turn, we-ness leads to “we-thinking” through which we see ourselves as similar to others in our group in an effort to normalize our behaviors (D’Errico et al., 2015). The term we-thinking refers to empathy that is based on positive inclinations to include those who are perceived to be like us, and destructive inclinations to exclude those who are dissimilar to us. It is necessary to understand the positive emotions resulting from we-ness as coping techniques to keep oneself from feeling mad, scared or sad. However, it is also necessary to understand that negative emotions can also be a result of marginality, as we will discuss in the next section.

##### Intergroup aggression

Intergroup aggression (i.e., intergroup conflict) is an altercation between two or more groups of people competing for values, resources, or rewards (Smith et al., 2015). Factors that can contribute to intergroup aggression include intergroup bias, we-thinking, and we-ness. Intergroup bias refers to people’s desire to see their group as superior to others, which can lead to conflict. Intergroup relations are framed by social identification models in which the individuals structure their perceptions of themselves and others by means of social categories. Members of a group internalize these categories as aspects of their self-perceptions and social-cognitive processes (Turner, 1982).

*Cyberterrorism.* Cyberterrorism is defined as an act that is motivated politically, socially, economically, or religiously to threaten or harm an outside party with the intent of creating fear or destroying assets using cyber systems (Stohl, 2006). Cyberterrorism stems from a rational choice evaluation in which the cost of taking action is low in comparison to the benefits (e.g., terrorists begin attacking via cyber systems as an efficient alternative to traditional [physical] actions; Stohl, 2014).

Cyberterrorism can be effects-based or intent-based. Effects-based terrorism is a form of hostile aggression in which the behavior is a reaction to being insulted or threatened, in perception or in reality (Stohl, 2006). The attacker’s motives are fear and ideology; attackers use ingroup and outgroup activity to prove how human interaction can lead to violence toward outgroup members. The actor aims to create fear, anxiety, and panic in the target population. By comparison, intent-based cyberterrorism is instrumental aggression that is initiated by the ideology and mastery needs of the actor. Intent-based terrorism exists when malicious attacks are employed to intimidate or force a group of people to change politically, to hurt them, or to impact their economic stability (Stohl, 2014).

*Discrimination.* Group-criterion is similar to we-thinking and we-ness, and establishes the traits by which an individual may be marginalized from the group. Discrimination, which is the basis of group-criterion, is a form of intergroup aggression and is defined as a group having a limited and identifiable set of traits that sets them apart from others (Thomsen, 2013). Discrimination occurs when an individual is treated unfairly compared to others in a similar situation because of their skin color, religion, disability, age, or sex (Teufl and Kraxberger, 2011). Discrimination in the form of “prejudice-related discrepancies,” can result from an individual automatically categorizing groups of people, also known as stereotyping (Frijda, 1996).

*Digital takedown (a.k.a. hacking).* A digital takedown is more commonly known today as hacking. Similar to effects-based cyberterrorism, cyber attackers normally promote hostile aggression. A number of online big data tools are being used to identify online malicious behavior. Social network analysis is a useful tool in identifying both malicious activity and the centralized individuals (nodes) who help direct or influence the malicious activity (Dimitrova et al., 2010; Lu et al., 2010). The strength of relationships between nodes may affect the propensity of a maliciously acting social group to execute a cyber attack.

#### Macro-Level of Interaction

Macro-culture refers to the mainstream culture of a large group such as a nation (Calori and Sarnin, 1991). The macro-level of analysis is important, because it allows us to examine general trends in cultural factors and how these trends affect overall cultural frames. National cultures, although subject to nuance at lower levels of analysis, provide widely accepted forms of socialization and help to determine how individuals may perceive maliciousness. In the context of cybersecurity, national cultures have produced major differences in framing cyber attacks. Further, the cultural framing of cyber attacks becomes more visible at the national level due to the vast array of information available. The national level also helps depict how maliciousness in cyber attacks is viewed differently than maliciousness in other realms; this section focuses solely on national attitudes toward maliciousness in cyberspace as we are able to better distinguish human maliciousness in the cyber realm from other forms. To understand the factors that have influenced these contrasting cultural frames we have employed a cross-cultural analysis. In this analysis, we highlight three nations, Russia, China, and the United States, over time, to understand how their contrasting laws, languages, contexts, and media interpretations of cyber attacks have prompted differences in the cultural framing of cyber attacks and how these frames may manifest as differences in the source, motivation, type, and purpose of attacks.

##### The United States

In the United States, there have been several variations in cultural frames of cyber attacks throughout time. Cyber attacks began with hackers in 1961; the Signals and Power Committee of MIT’s Tech Model Railroad Club obtained a PDP-1 (Programmed Data Processor-1), and from their interactions with this machine sprung an entire subculture around computer hacking. During the 1960’s, the term “hacking” took on a positive connotation and was believed to be a source of innovation in computer technology performed by highly skilled programmers (Yar, 2005). In the 1960s and 1970s, American hackers took on several aspects of the counterculture and used their work to promote the free flow of information and resist conventional authorities (Yar, 2005). However, hacking took a turn in the 1980s with the integration of personal computers (Clarke et al., 2003). As personal information became more readily available online, hacking became less about demonstrating computer knowledge and fostering innovation and more about obtaining another person’s confidential information and pirating software for personal gain. The film *WarGames* (Goldberg et al., 1983) depicted hacking as potentially disastrous and helped frame cyber attacks as harmful and destructive to society (Knapp, 2014). Shortly after *WarGames* was released, Congress enacted the Computer Fraud and Abuse Act (CFAA) in 1986, which provided severe punishment for certain types of cyber attacks (18 U.S.C. 1030, 2017). The legislation supporting the act referenced the movie *WarGames*, calling it a realistic depiction of the powers of personal computers. As the CFAA exaggerated the powers of personal computers, disdain for computer hackers increased in the United States (Knapp, 2014). *WarGames* and the reaction it elicited in the United States Congress is a clear instance of media’s ability to frame cyber attacks and influence actions regarding cybersecurity. The link between legal framing of cyber attacks and public perception is a feedback loop, exemplified by the passage of the CFAA and the subsequent increase in public worry and awareness.

However, as Americans’ perceptions surrounding hacking became increasingly negative, hackers began to use their skills for civil disobedience, a practice that is an integral part of political culture for many Americans (Knapp, 2014). The term “hacktivism” was then coined to describe the work of such activists who utilize cyber attacks to share their frustrations (Ruffin, 2004). Hacktivism in the United States has led many non-hackers to see the benefits of cyber attacks as a means of civil disobedience; supporters have even argued that provisions to hacking sanctions should be made to limit punishments for hacktivism (Knapp, 2014). Following the rise of hacktivism, Americans have begun to openly incorporate cyber attacks into military action. For example, in 2010, the United States and Israel (allegedly) built and deployed a worm called Stuxnet. The most advanced piece of malware to exist when introduced, Stuxnet was used to physically damage the nuclear centrifuges in an Iranian nuclear facility (Perlroth et al., 2013). Further, Stuxnet can be credited with highlighting global benefits of cyberwarfare, such as low cost and low casualties (Farwell and Rohozinski, 2011). These situations demonstrate that the cultural framing of cyber attacks varies based on the purpose of the attack, the target of the attack, and whether the attack aligns with societal values. Group values can be direct indicators of individual motives (Markus and Kitayama, 1991); therefore, cultural values may be important metrics associated human maliciousness in cyber attacks.

##### Russia

Russia has long fostered a wide array of culturally sanctioned cyber attackers. At least five historical and cultural factors have influenced Russia’s unique framing of cyber attacks, including: abundance of skilled computer scientists, relative lack of high-paying computer jobs, comparative acceptability of cybercrime in Russia, government sanctioning and use of hackers (especially in the last few years), and a common social disillusionment (Wilmes, 2006). In the 1980s, Russian hackers were sponsored by the Soviet government to pirate western software and adapt it to Russian computers; these attacks were widely deemed to be patriotic. From 1985 to 1991, Russia underwent Perestroika, the movement to reform the USSR. The collapse of the Soviet Union followed in 1991. This movement was accompanied by a more individualistic economy, and a more individualistic, optimistic, entrepreneurial culture that began to oppose central authority (Burke et al., 2015). In response to this movement, hackers wanted to provide more open information and software (Wilmes, 2006). Therefore, the way we perceive hacking or cyber attacks may be influenced by our proximity to a political or social revolution. The Criminal Code of the Russian Federation was introduced in 1996, somewhat criminalizing cybercrimes but giving cyber criminals less harsh punishments than other offenders (Criminal Code of the Russian Federation, 1996/2012). This legal framing of cyber attacks led many Russians to view them as less severe than other forms of crime (Wilmes, 2006).

After the Russian economic crises in 1998 and 2008, there was a return to authoritarianism and another significant cultural shift during which the Russian people were observed to become more cynical, materialistic, self-sufficient, nationalistic, and ethnocentric (Burke et al., 2015). The financial crises led many skilled programmers to lose their jobs, resulting in professional programmers becoming hackers for personal gain (Wilmes, 2006). Russian hackers described their motivation as centering on glory, fame, or revenge on the government. Therefore, economic factors and their effects on overall satisfaction with the government have historically been factors associated with performing malicious cyber attacks. In recent years, Russian cyber attacks have reflected the nationalistic, ethnocentric attitudes that resulted from the economic crises of 1998 and 2008. In 2007, Russian nationalists performed a Distributed Denial of Service attack on Estonia because Estonia planned to move a Russian war memorial. This attack is a clear demonstration of how cultural factors, such as international conflict and historical context, can influence individual perceptions of a situation and, in turn, influence individual hackers to act patriotically (Hathaway et al., 2012).

##### China

China is another nation that has performed, sponsored, or tolerated nationalistic hacking as a form of foreign cyber warfare or espionage. Much like the United States and Russia, China often considers cyber attacks a component of a larger, hybrid warfare strategy (Commin and Filiol, 2015; Lowe and Pitinanondha, 2015). In hybrid warfare, boundaries are blurred, resulting in greater engagement with the target country’s population. This involvement increases the likelihood of both attackers and targets incorporating elements of culture in their online behaviors. Cultural elements may involve the use of symbols, or linguistic phrases that imply values. In some cultures the rule “an eye for an eye” can extend beyond laws and customs into the online environment. The observations of Minkov (2011) and Morgan et al. (2015) of culture setting group norms, and learning occurring through imitation, would likely apply in this cyber environment.

Cultural values, including nationalism, patriotism, conflict resolution methods, and intolerance to new ideas, provide justification and explanation for malicious acts (Hofstede et al., 2010). This is the case with hacktivism in China, which is often characterized by pro-Chinese hackers and patriotism. The Honker Union, also known as the Red Hackers, is a Chinese hacktivist group; they abide by a strict code of conduct which states they must love and defend China against any defiant acts by foreign groups (Yip and Webber, 2011). In 2001, the United States and the Honkers Union engaged in a hacker war (Smith, 2001). The attacks were mostly cyber-graffiti, with hackers on both fronts defacing websites with their own nationalistic symbols and jokes. Social psychologists suggest that widespread Chinese nationalism and collectivist culture, coupled with the government’s national humiliation propaganda surrounding foreign threats, leads some hackers to act in the name of cultural pride and national security (Yip and Webber, 2011). Since the 2001 attack, China has allegedly employed hacking to steal United States information and technologies to bolster the Chinese economy; this is believed to be the case with the 2011 Night Dragon attacks during which China hacked five multinational oil and gas companies (Perlroth et al., 2013).

China’s recent international attacks support the idea that strong group affiliation often leads to high risk activism (Fine, 2012). Despite China’s widely sanctioned international cyber attacks, China has some of the most severe Internet censorship of any modern country (Xu and Albert, 2017). China manages to sustain constitutional freedom of press and speech as well as strict censorship through vague laws that allow authorities to both identify and outlaw “dangerous” information and block selective Internet sites and search engines (Xu and Albert, 2017). China’s selective characterization of specific activities as cybercrimes (i.e., use of dangerous information) demonstrates both culture’s role in justifying maliciousness and the maintenance of such justifications by legal framing and cultural norms. China’s widespread group values (nationalism, collectivism, and competition) may thus influence their motivations for cyber attacks.

### Developing Proposed Metrics

The goal of this research is to identify metrics that can be used to quantify cybersecurity risk originating from human factors interacting in the cyber realm. This paper is an attempt to bridge the gap between individual attributes and cultural influence contributing to personal behavior across both cyber and physical realms. Evaluating cyber-related maliciousness as a component of cyber activity is like peeling an onion: the exposed is what is perceived from the outside but as each layer is pulled away, a new layer is exposed. Peel back layers and you expose the core: who a person truly is without any extrinsic influences.

The metrics proposed to characterize maliciousness in this context have been analyzed before, but for different purposes. For example, social identification is the basis of all levels of interaction (interpersonal, meso, and intergroup) (Turner, 1982). If we relate the concept of social identification to known malicious behaviors, it is feasible determine the motivation driving the attack (Fluck, 2014). However, the majority of proposed maliciousness metrics weren’t specifically designed to evaluate maliciousness as much as simply evaluate human behavior, so we had to find relationships between concepts and results proposed by a diverse array of studies. Further, many of these factors do not have current applications to maliciousness research or methods of quantification. In terms of cultural research at the macro- and meso-levels of interaction, Hofstede’s work on cultural values has had the most notable applications. Hofstede’s cultural values have been studied in a variety of ways. They can be analyzed at a national level using demographics, history, politics and other national factors as we have done in the Section “Macro-Level of Interaction” of the present paper; they can also be analyzed at an individual level using surveys (Hofstede, 2011).

Individual assessment metrics were selected from the cross-disciplinary analysis on maliciousness using previously identified measures for a starting point. The metrics proposed for an individual’s malicious motivation were also partially determined through a cross-cultural analysis. The factors that created differing cultural frames, attack types, and frequencies in attacks were deemed important determinants of motives to perform malicious cyber attacks. For example, countries with state-sponsored attacks show significant cultural differences from countries that do not typically sponsor cyber attacks (Wilmes, 2006; Knapp, 2014). These disparities demonstrate that cultural values manifest as unique perspectives underlying the justification and motivation to perform a cyber attack. However, even though an individual’s culture may influence behavior, it’s ultimately the individual’s decision to act.

#### Proposed Assessment Metrics for Individuals

Personality traits and mental instability are the primary factors affecting the individual’s propensity to malicious action. Both personality traits and mental instability are quantified in the literature, often using survey approaches. However, at this point in time there has not been any direct linkage to whether or not a person is likely to behave maliciously, particularly within the cyber context. Personality traits of malicious individuals may be assessed using the theory of Dark Triad traits (Jones and Paulhus, 2014), possibly combined with Five Factor personality measure. Mental stability can be assessed using the Levenson Self-Report Psychopathy Scale, but there is a need for a scale to assess mental stress (Jakobwitz and Egan, 2006). Prior history is something that we believe has substantive importance as a disposition for behavior, and can easily be tracked by means of criminal records, social media, and interviews.

#### Proposed Micro-Level Assessment Metrics

Micro-level interactions can occur both online and in the physical realm, which offers more insight into how relationships affect behavior. Emotions may relate to mental (in)stability in that there is a range of emotional intensity, as mentioned previously. A person may be triggered to behave a particular way when their mental instability and emotions get out of control. There must be a way to quantify emotional intensity, which is defined by Bentley et al. (2014) as a function of: concerns, event appraisal, action repertoire, regulation and mood. Prior history as it related to future behaviors is also recognizable. Criminal records, newspaper stories, and social media accounts should and can be analyzed to depict whether or not a person seems like they may be triggered to behave maliciously in the future. Self-perception and attitudes can be assessed by simple questions to determine whether a person has optimistic or pessimistic tendencies. That being said, if a person is having a series of very intense emotions and also seems down on themselves or others, this should indicate a higher risk of behaving maliciously. The social cohesion model is used to assume each group member has a perception of themselves and others by means of “abstract social categories,” and they their self-concepts are a basis of this. Social cohesion model has large-scale categorical use, such as race, sex, and nationality (Turner, 1982). Lange and Crusius (2015), completed a study using Amazon Mechanical Turk to relate envy to maliciousness tendencies. They ran four different studies to first, determine the difference between malicious and benign envy, observe how hope and success and fear of failure relates to malicious and benign envy, and then to analyze the ways in which behavior changes the envy an individual undergoes. They found behavior and motivation are correlated with both malicious and benign envy. Envy can be related to attitudes in that if a person feels as if they need to bring others down to raise themselves, they may behave maliciously to do so. Biases, as examined by LoBue et al. (2010), can be recognized by learning experiences. Analyzing children versus adult behavior is a way to identify and classify certain biases toward events or objects. Lastly, marginality is something that we may have to look into history for. Marginality is a subject that many people don’t like to discuss. For example, African Americans (especially poor African Americans) have be marginalized throughout United States history and to this day. Ethnicity, gender, sex, household income, education, and other factors may contribute to the marginality of an individual.

#### Proposed Meso-Level Assessment Metrics

When examining meso-level influences to act maliciously or, more specifically, perform malicious cyber attacks it is important to incorporate both general and cyber-specific factors for joining deviant peer groups. In terms of the general factors associated with participation in malicious-acting, deviant groups we have identified: low socioeconomic status, hyperactivity, limiting one’s social behavior to the peer group, family adversity, being of the male gender and socialization toward rule-breaking behavior, both on and offline (Lacourse et al., 2006). Further, hackers have helped to identify a number of factors associated with participating in malicious-hacking groups; these factors are: smart children’s boredom with inadequate schools, schools’ inability to notice the children’s differences, and the desire to act out against corporations that profit from overpriced web services (Blankenship, 1986; Yar, 2005). Also, participation in particularly misogynistic hacking groups is often associated with disdain or frustration with women (Yar, 2005). Additionally when the larger culture associates masculinity with computer-related skills, cyber attackers may join deviant hacking groups as a means to assert their masculinity (Yar, 2005). Beyond deviant peer groups, one must incorporate meso-level cultural values as well as intergroup behavior to better understand what may lead individuals within subcultures to act maliciously.

#### Proposed Cultural Motivation Assessment Metrics

Proposed cultural metrics draw from both the work of Hofstede and the cultural factors that influence cultural and perceptual variances in the cross-cultural analysis of the United States, China, and Russia; we believe these factors have historically been associated with cyber attacks.

The cross-cultural comparison above demonstrated the importance of the current state of the economy and the government in the propensity of an individual to conduct cyber attacks. In times of poor economic conditions, revolution, or overall dissatisfaction with the nation, there have been increases in cyber attacks; thus, national political and economic stability may influence social perceptions that promote individual actions. Therefore, the unemployment rate, national GDP, average satisfaction with government, type of government, and political stability are probable cultural factors to explore for inclusion in maliciousness metrics (Burke et al., 2015).

The media’s depiction of cyber attacks also plays a role in the way citizens perceive attacks (Knapp, 2014). The legal status of cyber attacks and the government’s relationship with cyber attacks are important, given that the legal framing often guides individual perceptions of hacking and governmental sponsoring of cyber attacks has bearings on the expertise and favorability of hackers (Wilmes, 2006). Intergroup behavior, as evidenced by multiple acts of cyberwarfare and competition between groups, also contribute salient cultural factors on both the meso- and macro-levels (Yip and Webber, 2011) Intergroup behavior will likely be assessed through self-reports of perceived intergroup conflicts of interest, as these perceptions are often direct indicators of intergroup aggression (Struch and Schwartz, 1989). It is also important to measure both the degree to which an individual favors the in-group as well as the strength of their group affiliation as both of these traits can lead an individual to support in-group bias and intergroup aggression (Fine, 2012).

Finally, cultural values play an integral role in measuring the meso- and macro-cultural factors that contribute to cyber attack behavior. Cultural values include patriotism, civil disobedience, censorship, and military success (Hathaway et al., 2012).

Many of these metrics have established and publicly available forms of quantification, such as unemployment rate and national GDP. For some metrics, a means of quantification is currently lacking. Among these metrics are the legal status of cyber attacks, the governmental relationship with cyber attacks, and the media’s depiction of cyber attacks. Legal status requires investigation of a country’s laws, while the latter two metrics could be measured via literature analysis and computational linguistics.

##### Hofstede’s cultural dimensions

**FIGURE 4:**
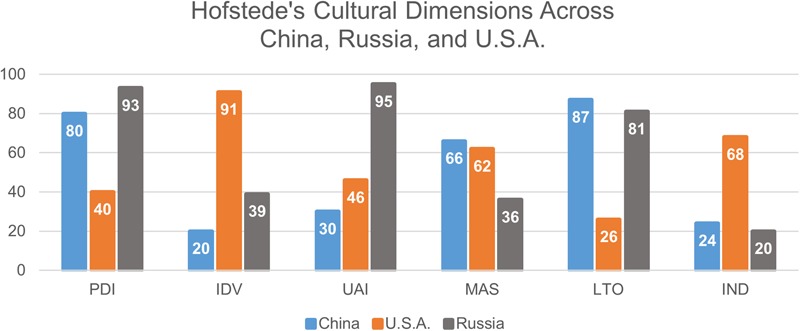
Hofstede’s dimensions across China, Russia, and the United States. This graph depicts the differences and similarities between the aforementioned countries in terms of their cultural values as defined by Hofstede (Hofstede, n.d.). Uncertainty avoidance index (UAI) signifies a culture’s tolerance for ambiguity; the more comfortable a group is with ambiguity the lower it scores on this scale. Masculinity vs. femininity (MAS), or the preference for power or nurture in a group, demonstrates the dynamic between these traits by assigning low scores to feminine and nurturing groups. Long-term orientation vs. short-term orientation (LTO) describes a group’s goal orientation; low scores on this scale mean a group has short-term goals. Power distance index (PDI) or the distribution of power within a society; lower scoring societies are more egalitarian. Also, indulgence vs. restraint (IND) show the degree to which group members indulge; low-scoring cultures are less indulgent. Individualism vs. collectivism (IDV) measures a group’s values of group solidarity. More collectivist cultures score lower.

To provide a more comprehensive assessment of these cultural values, we will use the six cultural dimensions of Hofstede: power distance, uncertainty avoidance, individualism vs. collectivism (IDV), masculinity vs. femininity (MAS), long-term vs. short term orientation of choice, and indulgence vs. restraint (IND) (Hofstede, 2011). Power distance index (PDI) relates to the distribution of power within a society; the lower the score on this scale, the more egalitarian a society is. Uncertainty avoidance index (UAI) measures a culture’s tolerance for ambiguity as determined by the amount of rules a society has; the more comfortable a society is with ambiguity about the future, the less rules they have, and the lower they score on this scale. IDV measures a group’s values of group solidarity. The lower the score on this scale, the more collectivist a culture is. Hofstede also incorporates MAS to describe the preference for power or nurture in a society; groups that receive low scores on this metric are more feminine and nurturing. Long-term orientation vs. short-term orientation (LTO) focuses on the prominent goal orientation of a group; low scores on this scale signify a propensity for short-term goals. Finally, IND highlight the degree to which group members indulge; cultures that receive low scores on this metric are less indulgent than high scorers. **Figure 4** provides an example of Hofstede’s metrics, graphing the results for China, Russia and the United States. China and Russia are ranked similarly on four of Hofstede’s six metrics (PDI, IDV, LTO IND), while the United States is most similar to China in UAI and MAS. By comparison, the United States and Russia seem dissimilar for virtually all the metrics. Future analysis may demonstrate that these factors correlate with government-sponsored attacks, or societies that often perform government-sponsored attacks.

## Discussion

Our research outlining the potential metrics for human maliciousness in the cyber realm is the first time cybersecurity researchers have attempted to gain a holistic image of humans as malicious actors in the cyber realm. In quantifying cybersecurity risk, researchers have been able to quantify expertise, insider threat, and (economic) rationality but metrics and methods to quantify human maliciousness as a cyber risk factor has been lacking. Because there has been little research on human maliciousness in the cyber realm, we have looked at potential sources of maliciousness metrics outside cybersecurity studies where human maliciousness has been examined at the individual, micro, meso, and macro-level. All of the factors likely contribute to ultimate intent to harm within the cyber realm. Individuals may act maliciously due to personality, but their behavior is still influenced by their interactions with others. Group membership and intergroup aggression may contribute to an individual’s motivation to behave maliciously, as may cultural biases, which help guide our perceptions of cyber attacks and, in turn, influence us to behave in specific ways. Maliciousness is a sociotechnical issue, and it is important to determine the optimal metrics to use when integrating human factors into cybersecurity risk assessment. And after much consideration of the literature, we conclude that people’s thoughts and behaviors are equally important to malicious code in the exploitation of vulnerabilities in technology.

### Gaps in Research

Our work presents a holistic picture of human maliciousness within the context of cybersecurity, but we acknowledge that our research may be incomplete. The following sections address some of the limitations of the research we have uncovered.

#### Limitations of the Individual Maliciousness Literature

The largest problem with respect to classifying cyber attackers on an individual basis is that there is a lack of data on malicious behavior specifically. Various tests measure traits, characteristics, motivations, values, and biases associated with behavior, such as the Mach-IV (Jakobwitz and Egan, 2006), York Enviousness scale, (Lange and Crusius, 2015), the Levenson Self-Report Psychopathy Scale (Jasper, 2015), Elaboration Likelihood Model (Petty et al., 1986), and Confirmatory Factor Analyses (Fluck, 2014). Some of these assessment tools are starting to be used to connect maliciousness-associated behaviors with cyber threat activity; for example, Whalen and Gates (2007) implemented the International Personality Item Pool Representation of the Revised NEO Personality Inventory (NEO-PI-R) survey design to predict the likelihood of insider threat. The semi-quantitative test they used to identify personality traits consisted of 120 questions, each measured on a five-point Likert scale. This 120-question test is half as long as the 240-question Five Factor (NEO-FFIR-R) inventory. Despite progress toward detecting characteristics contributing to maliciousness, little has been done to develop and test a reasonably sized survey or other instrument that can be used to test multiple malicious-related metrics in the same population. Rather, assumptions inferring motivation and intent are drawn indirectly from behavior, making these endpoints more difficult to quantify and validate. It is also difficult to assess the error and uncertainty in the metrics.

#### Shortcomings in Micro-Level Literature

The micro-level literature focuses on interactions between people. However, interactions are often dictated by circumstantial factors such as the personalities, beliefs, sociometric status, and attitudes of the humans involved, the culture of the individuals, and the situation in which they find themselves. It is challenging to create a complete list of micro-level factors when human interactions are very situational, and difficult to study. Further, the emotions that arise out of our interactions with others can be very hard to gauge both for researchers observing human behavior as well as the humans experiencing these emotions. No one has yet formed a comprehensive index of the emotions associated with maliciousness. It is also difficult to obtain complete information about how an individual perceives themself as well as their attitudes and biases. Thus, our understanding of more covert human behaviors that occur at the micro-level may be too limited at this time to use these metrics as part of a set of metrics to assess cyber maliciousness. However, the above identified studies provide a starting point for determining future micro scale human maliciousness metrics once additional fundamental research is completed.

#### Shortcoming in the Meso- and Macro-Level Literature

The literature on the cultural framing of cyber attacks is limited. To gain a holistic view of the factors associated with cyber attacks and malicious behavior one would have to survey attackers about their culture and how it has influenced their online behavior; research of this nature is scarce. Much of the literature relies upon deductions about factors associated with cyber attacks based on variations between cultural framing and the types of attacks these frames elicit. There could be inaccuracies regarding the cultural factors that influence individual maliciousness. Additionally, while literature regarding deviant peer groups is abundant, its application to the cyber realm is scarce. Our knowledge about hacking subcultures within different countries, therefore, is limited. Furthermore, many people may view the assignment of cultural characteristics as a form of profiling. To assume that any given individual within a group will be associated with the same metrics is undoubtedly problematic. However, combining cultural and individual metrics should help account for differences between the individual and their culture.

### Future Direction of Research

It is hard to characterize maliciousness, and harder yet to develop a maliciousness index to apply to models of human factors and human behaviors using standard psychological testing. These malicious behaviors are not always manifest and are less likely to manifest during such overt testing; and while they can be simulated using gaming environments, the results are not likely to be true characterizations of someone acting at the level of maliciousness of a terrorist attack or a cyber attack that truly harms many people. Further, truly malicious people would likely try to subvert any testing that is aimed at characterizing maliciousness. Therefore, maliciousness may be assessed (as one approach) by evaluating naturally observable language through communications that are spontaneously generated for other purposes (web pages, Twitter, blogs). Building an ontology based on the taxonomy and flowchart helped establish and categorically organize relationships between culture and individual personality characteristics, both of which influence the way a person behaves and thus maliciousness. Additional research is needed applying that ontology to tease out the factors that most contribute to cyber-linked maliciousness. At this time, the research will need to be targeted to characterize the influence of one or a few related metrics at a time, but future work could integrate the fundamental correlative metric research and build a more complex model across individual, micro, meso and macro factors. In attempt to further study a more narrowly tailored set of metrics, immediate future research will be focused on the effects of personality and culture on the prevalence and acceptance of deviant behaviors. This research will feature surveys, distributed to college students in several different countries, which will analyze the interplay between malicious personality traits, namely the Dark Triad, and Hofstede’s cultural values; it will also measure the degree to which values and personality are associated with online deviance. Any definitive model to assess the risk associated with malicious cyber behavior is that both the culture and motivation metrics will need to be integrated to better understand how an individual is motivated by extrinsic and intrinsic influences. We expect such a model will, at the least, identify patterns in the linkage between cybersecurity-related behavior, motivation and culture.

## Author Contributions

ZK wrote the culture and group pieces, developed the framework for motivations, wrote introduction paragraphs, edited and the rewrote sections. DH oversaw and coordinated the work going into the paper, wrote parts of the paper (especially introduction), edited the whole paper and rewrote sections. LF contributed literature regarding an individual’s propensity to inflict harm upon others. MC contributed to the conceptual framing of the research topic in addition to paper organization and editing. BH contributed sections on cyber and cyber attacks and provided cyber expertise, feedback, and edits. CS contributed cultural and cyber area expertise.

## Conflict of Interest Statement

The authors declare that the research was conducted in the absence of any commercial or financial relationships that could be construed as a potential conflict of interest.
